# Cold atmospheric plasma: A non-negligible strategy for viral RNA inactivation to prevent SARS-CoV-2 environmental transmission

**DOI:** 10.1063/5.0060530

**Published:** 2021-08-10

**Authors:** Tao Jin, Yong Xu, Chenwei Dai, Xiuhong Zhou, Qinghua Xu, Zhengwei Wu

**Affiliations:** 1 School of Nuclear Science and Technology, University of Science and Technology of China, Hefei, China; 2 Anhui Academy of Medical Sciences, Hefei, China; 3 Key Laboratory of Geospace Environment, Chinese Academy of Sciences, Hefei, China

## Abstract

Cold atmospheric plasma (CAP), regarded as a powerful physics technology, displays antimicrobial, antitumor, and even antiviral properties, but the underlying mechanism is rarely studied. In this study, four CAP exposure doses (30, 60, 120, and 240 s) were applied to inactivate a severe acute respiratory syndrome coronavirus 2 like pseudovirus on a stainless steel disk, which comprised spike protein on its membrane and can express a green fluorescent protein. In order to unravel the potential effects of CAP irradiation on pseudovirus, infection assay, optical emission spectra analysis, transmission electron microscopy (TEM), sodium dodecyl sulfate polyacrylamide gel electrophoresis, ELISA, and qPCR experiments were carried out. As a result, our study indicated that CAP irradiation can significantly decrease the infectivity of pseudovirus in a dose dependent manner through destroying the cell membrane and further damaging viral RNA, with the molecular weight and conformation of spike receptor binding domain protein unchanged.

## I. INTRODUCTION

At the end of 2019, COronaVIrus Disease-2019 (COVID-19) was first reported in Wuhan city, China, and gradually developed into a global pandemic.^1,2^ Severe acute respiratory syndrome coronavirus 2 (SARS-CoV-2) is the pathogen of COVID-19, and thus suppressing its transmission is regarded as the key step to control and decrease COVID-19.^3^ Unlike SARS-CoV-1 (in 2002) and Middle East respiratory syndrome coronavirus (MERS-CoV, in 2012), SARS-CoV-2 showed lower mortality (2.08%) but a higher infectious rate (2.23%) until May 29, 2021 due to its fast propagation speed (https://www.who.int/emergencies/diseases/novel-coronavirus-2019). SARS-CoV-2 is composed of 16 non-structural proteins (ORF1ab, ORF3a, ORF7, ORF8, etc.) and four structural proteins, including spike (S) protein, membrane (M) protein, envelope (E) protein, and nucleocapsid (N) protein.^4–6^ The receptor binding domain (RBD) of S protein is mainly responsible for recognizing and binding with a human angiotensin-converting enzyme 2 (hACE2) receptor so that S protein plays a critical role in cell infection.^7^ Since the Chinese researchers announced the full RNA genomic sequences of SARS-CoV-2 on January 10, 2020, every research institution, medical company, university, and hospital around the world started to develop vaccines.^8–11^ Until May 7, 2021, there were six candidate vaccines for emergency using lists (EUL), including Pfizer/BioNTech (BNT162b2), AstraZeneca-SK Bio (AZD1222), Serum Institute of India, Janssen (Ad26.COV2.S), Moderna (mRNA-1273), and Sinopharm COVID-19 vaccine released by WHO (https://www.who.int/news/item/07-05-2021-who-lists-additional-covid-19-vaccine-for-emergency-use-and-issues-interim-policy-recommendations). Moreover, there also are more than 100 vaccines under the phase I/II/III/IV clinical trials.^12,13^ On the other hand, many researchers focus on repurposing FDA authorized drugs, such as remdesivir (against MERS and SARS), hydroxychloroquine (HCQ, an antimalarial drug), or lopinavir–ritonavir (treat HIV/AIDS) combination against SARS-CoV-2, and the results indicated that these drugs not only alleviate the symptoms (cough, taste disorder, myalgia, fever, dyspnea, headache, etc.) but also promote recovery.^14–18^

Although there have been many vaccines and effective agents against SARS-CoV-2, further evaluation of their ability to inhibit the variants of SARS-CoV-2, including B.1.1.7 (British variant), 501Y.V2 (South African variant), and P.1 (Brazil variant), is needed.^19–21^ On the other hand, we should rethink and pay attention to the environmental transmission of SARS-CoV-2.^22,23^ Latest news and research reported the transmission of SARS-CoV-2 via direct contact between humans and objects (plastic and stainless steel, etc.) whose surface was contaminated by the respiratory droplets of an infected person.^24^ The results of Meyerowitz *et al.*^25^ and Carraturo *et al.*^26^ pointed out that the viral RNA is still viable on plastic and stainless steel for a long time (more than 72 h). Numerous cases also reported environmental transmission of SARS-CoV-2 through wastewater, fecal discharges, droplet spray, and aerosols in universities, hospitals, prisons, etc.^27–29^ Thus, it is emergency to take some effective actions to prohibit environmental transmission. Tan *et al.* have mentioned that chlorinated disinfectants, 75% ethanol, and heat showed effective inactivation of SARS-CoV-2.^30^ Criscuolo *et al.* also presented that ozone (4 ppm) exposure for 120 min led to 98.2%, 99.8%, 99.8%, 90%, and 93.3% viral titer reduction of SARS-CoV-2 on glass, gauze, sanitize fleece, plastic, and wood surface, respectively. While UV-C light (1.8 mW/cm^2^) irradiation for 15 min displayed an absolute antiviral property (>99.9%) on glass, plastic, and gauze; 90% and 94.4% titer decrease on fleece and wool, respectively.^31^ Moreover, Biryukov *et al.* indicated that high temperature (54.5 °C) heat for 35.4 ± 9.0 min could shorten the half-life of SARS-CoV-2 and lead to 90% reduction in infectivity.^32^ Even though there are many effective physics methods (UV-C light, ozone, high temperature, etc.) to inactivate SARS-CoV-2 on different materials, most of them require long duration and energy consumption and even present side effects on the human body and environment.^33–36^ Thus, a more effective, economical, without side effects, and green mean to avoid environmental transmission of SARS-CoV-2 is urgent.

Plasma is a kind of ionized gas (contains ions, free radicals, neutral particles, electrons, and ultraviolet rays), which is also regarded as the fourth state of matter.^37^ In the past few decades, cold atmospheric plasma (CAP) was widely used in antibacterial, antiviral, anti-inflammatory, and even antineoplastic fields due to its powerful, friendly, inexpensive, safe, and green features.^38–40^ We looked for original research about SARS-CoV-2 inactivation through Web of Science, ScienceDirect, and Wiley Online Library using the topics “SARS-CoV-2 inactivation,” “cold atmospheric plasma inactivates SARS-CoV-2,” “nCoV-19 inactivation,” and “SARS-CoV-2 environmental transmission.” As a result, there were no studies reported using cold atmospheric air plasma to treat SARS-CoV-2 and even pseudovirus.

Finally, taking account of the assays related to SARS-CoV-2, including culture and infection, studies should be carried out in biosafety level 3 (BSL-3) laboratory.^41^ On the other hand, numerous base studies about the antiviral property of different chemical and physics methods on the fragments of real SARS-CoV-2 are equally necessary. Thus, in this study, we used a self-made dielectric barrier discharge (DBD) plasma device to treat a SARS-CoV-2 pseudovirus on a stainless steel disk. The pseudovirus comprises a short strand RNA that could transcribe and translate into green fluorescent protein (GFP), and its phospholipid envelope was covered by abundant S protein of SARS-CoV-2. The infection ability, spike RBD protein, and RNA changes of plasma treated pseudovirus were investigated.

## II. MATERIALS AND METHODS

### A. Reagents

The pseudovirus was purchased from Fubio Co., Ltd. (China). The spike RBD protein was obtained by Peptides Co., Ltd. (China). The complete culture medium (CCM) was prepared with 89% DMEM, 10% fetal bovine serum, and 1% penicillin streptomycin, which were obtained from Hylone Co., Ltd. (USA), Gibco Co., Ltd. (USA), and Beyotime Co., Ltd. (China), respectively. While the human embryonic kidney (HEK) 293T cell was designed by Fubio Co., Ltd. The Coomassie bright blue R250 staining fluid (CBB) was prepared with 0.1% Coomassie blue, 40% ethanol, 10% acetic acid, and 50% deionized water.

### B. CAP treatment

**FIG. 1.**
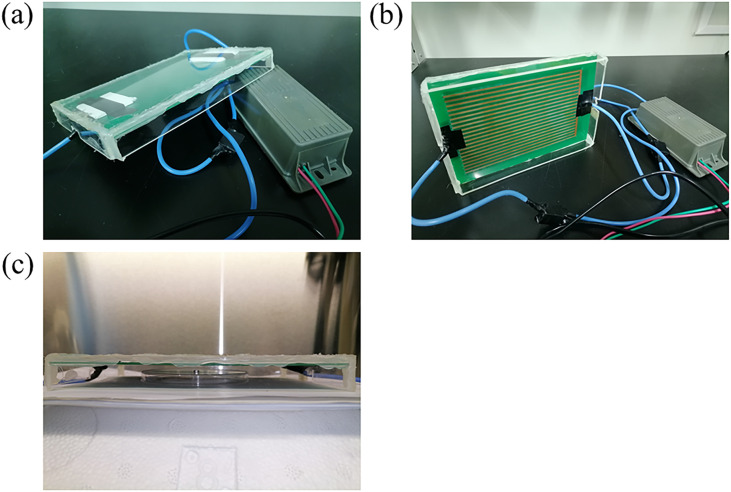
The schematic diagram of the DBD device (a) and (b) and sample treatment (c).

As shown in Figs. 1(a) and 1(b), the DBD device (10 * 15 cm^2^), also called the creepage discharge device, is designed and manufactured by ourself. It belongs to one of the cold atmospheric plasmas (CAPs) because it works at atmosphere and the feed gas is air. The output voltage, current, and frequency of the DBD device are around 8 kV, 40 mA, and 20 kHz, respectively. In order to make the reactive species (RS) produced by plasma interact enough with pseudovirus samples and get rid of the impact of flow air, we added a 2 cm wide PMMA board to seal the four sides of the DBD device. As shown in Fig. 1(c), 8 *µ*l of 2 × 10^7^ TU/ml pseudovirus was deposited on a stainless steel disk (diameter = 5 mm) and then covered by DBD devices. Moreover, a Petri dish was used to support the sample to make sure that there is 5 mm distance from the discharge area. Finally, the pseudovirus sample was dried in a biosafety cabinet for 30 min and then exposed to CAP for 30, 60, 120, and 240 s, coded as C30, C60, C120, and C240, respectively. The untreated pseudovirus was set as normal control and abbreviated as NC.

### C. Pseudovirus infection assay

**FIG. 2.**
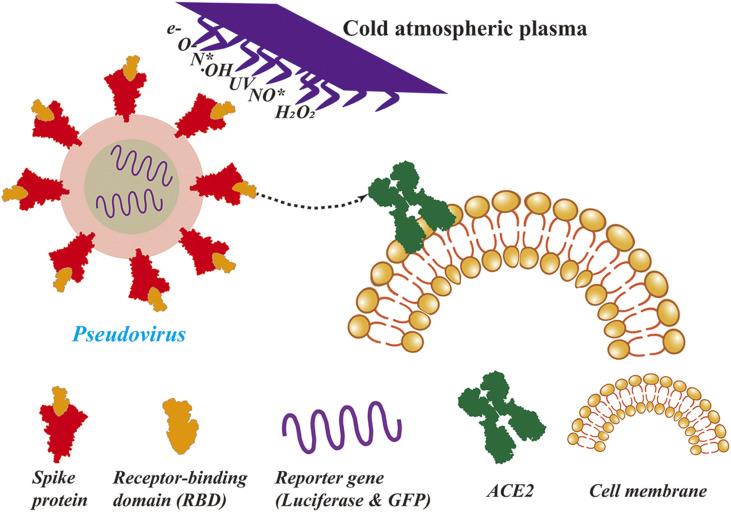
The schematic diagram for the pseudovirus infecting 293T cell.

As shown in Fig. 2, the schematic diagram for the pseudovirus infecting 293T cell is straightforwardly described. The infection experiment was carried out to investigate the effect of CAP irradiation on the infection activity of pseudovirus. The host cell used in this experiment was a kind of transformed HEK 293T cell, which can highly express the ACE2 receptor. The viral RNA of pseudovirus will translate into GFP when it enters the 293T cell. Thus, the infection ability of pseudovirus can be measured based on the green fluorescence intensity. First, the dried and CAP treated pseudovirus was eluted with CCM five times from the stainless steel disk, and 120 *µ*l of pseudovirus containing medium was obtained. Second, the 293T cells were added into a 96-well plate filled with 120 *µ*l of CCM and incubated under 37 °C and 5% CO_2_. The residual CCM in the plate was removed when the 293T cell grew to 40% of the area, and then 120 *μ*l of pseudovirus containing medium was added in to infect the cell. Similarly, the residual pseudovirus containing medium was completely removed after 7 h of infection, and another fresh 120 *μ*l of CCM was added into the wells. Finally, the green fluorescence intensity of each group was observed under an inverted fluorescence microscope (Leica, Germany) at 200× magnification after incubation for 72 h.

### D. Optical emission spectra

In order to confirm the RS in the DBD plasma system, the optical emission spectra (OES) information of the DBD plasma was characterized by an AvaSpec-2048 spectrometer. The probe of the charge coupled device (CCD) camera was placed under the DBD board, and the distance was controlled as 5 mm [the same place as treated samples in Fig. 1(c)]. Finally, the result was averaged by the values of three parallel experiments.

### E. Transmission electron microscope

In the morphology observation assay, 2 *µ*l of 2 × 10^7^ TU/ml pseudovirus was diluted 30 times with phosphate buffered saline (PBS) and then dropped onto a copper screen (230 meshes). The samples were stained with 2% uranyl acetate and dried under a tungsten lamp (40 W) for 15 min and then treated by CAP for 0, 30, 60, 120, and 240 s. Finally, the visual field to under-focus through Fourier transform calibration system was adjusted, and the viral morphology in different groups was observed at 43 000 magnifications by a 120 kV transmission electron microscope (FEI, USA).

### F. Spike RBD protein measurement

According to the previous research, the infection procedure of SARS-CoV-2 starts with the recognition of the hACE2 receptor on the host cell by the spike RBD protein.^42^ Thinking about this natural characteristic of cell entry, the molecular weight and conformation changes of spike RBD protein treated by CAP were investigated by sodium dodecyl sulfate polyacrylamide gel electrophoresis (SDS-PAGE) and ELISA, respectively. 5 *µ*l of 1 mg/ml spike RBD protein, obtained by diluting 2.5 mg/ml initial protein solution with PBS, was dropped on the stainless steel disk. The spike RBD protein samples, which were dried for 20 min in a biosafety cabinet, were exposed to CAP for 30, 60, 120, and 240 s, coded as C30-P, C60-P, C120-P, and C240-P, respectively. The untreated spike RBD protein was set as the control group and abbreviated as NC-P. The spike RBD protein treated by CAP was eluted with PBS five times, and finally 120 *μ*l of spike RBD protein containing PBS were obtained.

#### 1. SDS-PAGE

First, the solution, prepared by mixing 8 *µ*l of the spike RBD containing PBS with 2 *µ*l of 5× loading buffer, was heated in boiling water for 5 min. Next, the mixture solution was cooled to room temperature and loaded into a 15% PAGE gel. In order to intuitively compare the molecular weight changes of spike RBD protein in each CAP treated group, a protein marker in the molecular weight range from 10 to 180 kD was used as a reference. Finally, the SDS-PAGE assay was carried out at 200 V for 70 min, and the gels were stained by CBB.

#### 2. ELISA

The ELISA experiment was conducted following the manufacturer’s instructions of the SARS-CoV-2 spike protein S1 RBD ELISA kit. First, 20 *µ*l of spike RBD protein was added into a 96-well plate and incubated for 1.5 h at 37 °C. Second, the supernatant liquid was discarded and 100 *µ*l of biotinylated anti-SARS-CoV-2 S1 RBD antibody was added into each well to get interact with spike RBD protein for 1 h at 37 °C. Whereafter, each well was washed using wash buffer solution three times and incubated with 100 *µ*l of horseradish peroxidase labeled with avidin for 30 min at 37 °C. Finally, all wells were washed five times and then 90 *µ*l of tetramethylbenzidine (TMB) and 50 *µ*l of stop solution were added into each well. The optical density (OD) value of each group was measured at 450 nm by a microplate spectrophotometer (Bio-Rad, USA).

### G. RNA determination

SARS-CoV-2 virus is a novel and contagious coronavirus emerged at the end of 2019, which has seriously threatened human healthy and public safety.^43^ Thus, in order to explore the effect of CAP irradiation on the RNA of pseudovirus, the concentration of RNA in NC, C30, C60, C120, and C240 groups was detected. Total viral RNA and cDNAs were obtained according to the manufacturer’s protocol of the Tianamp virus RNA kit (Tiangen, China) and Fastking RT kit (Tiangen, China), respectively. The primers of *luciferase* used in this experiment were purchased from BGI Genomics and its sequences are shown as follows:

*luciferase* (Forward): 5′-CGCCATTCTACCCACTCGAA-3′,
*luciferase* (Reverse): 5′-CCGAACGCTCATCTCGAAGT-3′.

Finally, the qPCR assay was conducted by a real-time qPCR system (7500, ABI, USA), while the reaction solutions and time setting are listed in Tables I and II. The relative concentration of cDNA and RNA in each group was calculated by the ratio of 2^−ΔC^.

**TABLE I.** Solutions used in the qPCR system.

Name	Volume (*μ*l)
2× SuperReal PreMix Plus	25
50× ROX reference dye	1.0
Forward primer (10 *µ*M)	1.5
Reverse primer (10 *µ*M)	1.5
Template DNA	2.0
RNase free water	9.0
Total	40

**TABLE II.** The reaction time setting of the qPCR system.

Temperature (°C)	Time	
95	15 min	
95	10 s	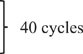
60	32 s	
60	60 s	

### H. Statistical analysis

All experimental data were recorded and calculated by Excel 2019. The significant difference with each group was analyzed by IBM SPSS statistics 21. All figures were plotted using Origin 8.5, Adobe Illustrator 2020, and Image J. All assays were operated at least three times, and final values were written as mean ± SD.

## III. RESULTS

### A. Pseudovirus infection activity

**FIG. 3.**
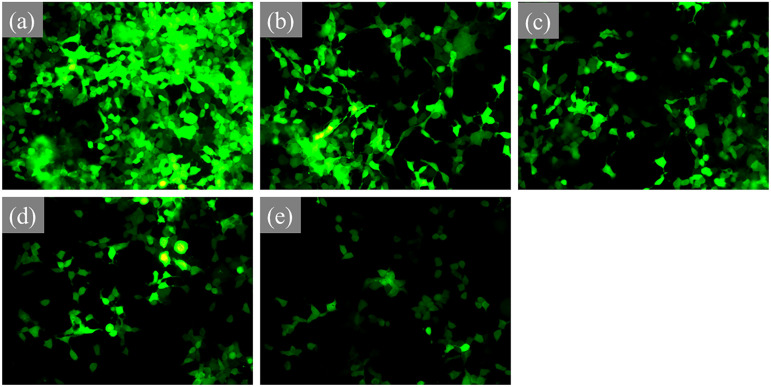
The viral infection assay: fluorescent image of GFP in NC (a), C30 (b), C60 (c), C120 (d), and C240 (e) groups.

The pseudovirus infected 293T cell can express GFP so that the green fluorescence intensity of each group was measured to evaluate the effects of CAP treatment on pseudovirus infection activity. As shown in Fig. 3, the 293T cell in the NC group displayed strong green fluorescence in a defined area, while the fluorescence intensity in CAP treated groups was significantly decreased. Furthermore, the intensity of green fluorescence in C240 was deeply reduced and it was hard to find any green spots in the image [Fig. 3(e)].

### B. OES

**FIG. 4.**
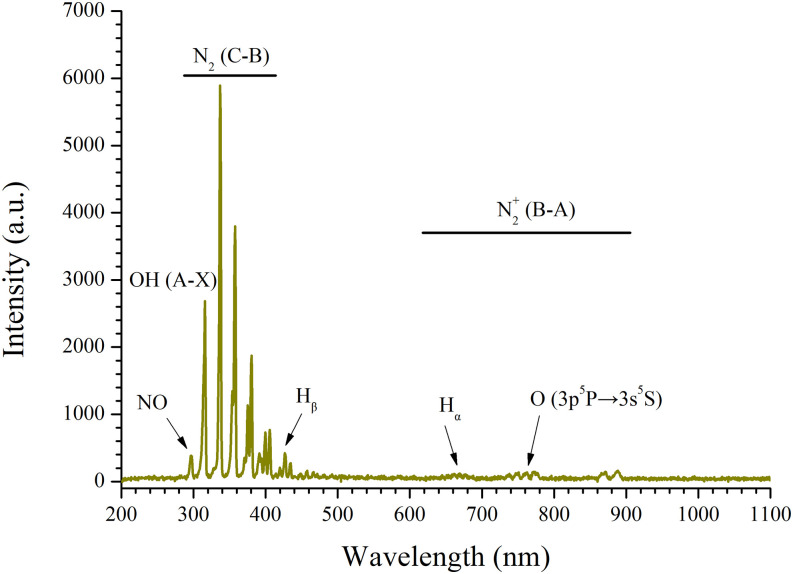
Optical emission spectra (200–1100 nm) of the air feed DBD plasma.

Figure 4 presents the emission spectral characteristics of the RS in the DBD plasma system in the spectral range from 200 to 1100 nm. As it can be seen in the range of 340–410 nm, there are some obvious peaks, including λ = 340, 359, 381, and 401 nm, which were attributed to the emission of the second positive system of molecular nitrogen N_2_ (C-B). On the other hand, in the range of 620–910 nm, there are many feeble peaks related to the first negative system of molecular nitrogen ions N_2_^+^ (B-A) located at the wavelength band from 620 to 910 nm. The peak at λ = 772 nm corresponds to the oxygen atom transition O (3p^5^P → 3s^5^S). Second, the OH (A-X) emission signal can be isolated from the wavelength λ = 313 nm.

### C. Morphological image of pseudovirus

**FIG. 5.**
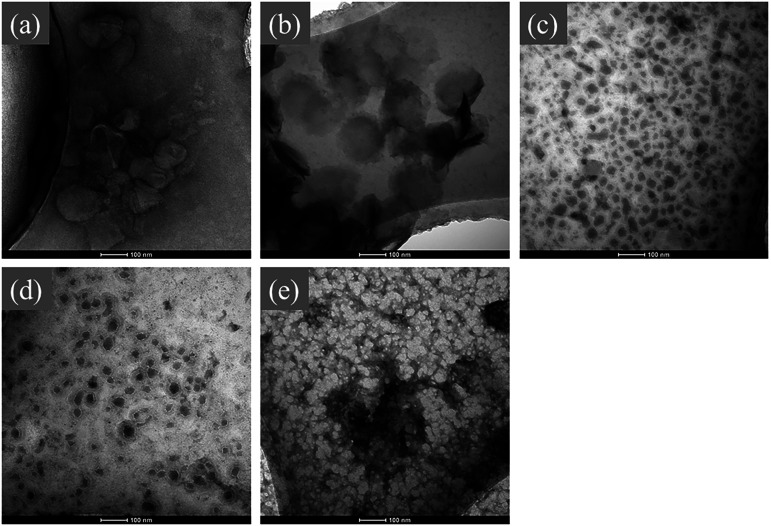
The viral morphology of pseudovirus: NC (a), C30 (b), C60 (c), C120 (d), and C240 (e) groups (plotting scale: 100 nm).

Figure 5 shows the viral morphology of the CAP treated pseudovirus in NC, C30, C60, C120, and C240 groups. The structure of pseudovirus in both NC [Fig. 5(a)] and C30 [Fig. 5(b)] groups was intact, while some wrinkled virus particles can be observed in the NC group. Furthermore, as shown in Figs. 5(c) and 5(d), it is easy to infer that the uranyl acetate dye completely entered the pseudovirus in C60 and C120 groups. However, the counts of the stained virus particles in C120 were less than that compared with the C60 group. As shown in Fig. 5(e), there were no any intact virus, but abundance of viral fragments were observed.

### D. The effect of CAP treatment on spike RBD protein

**FIG. 6.**
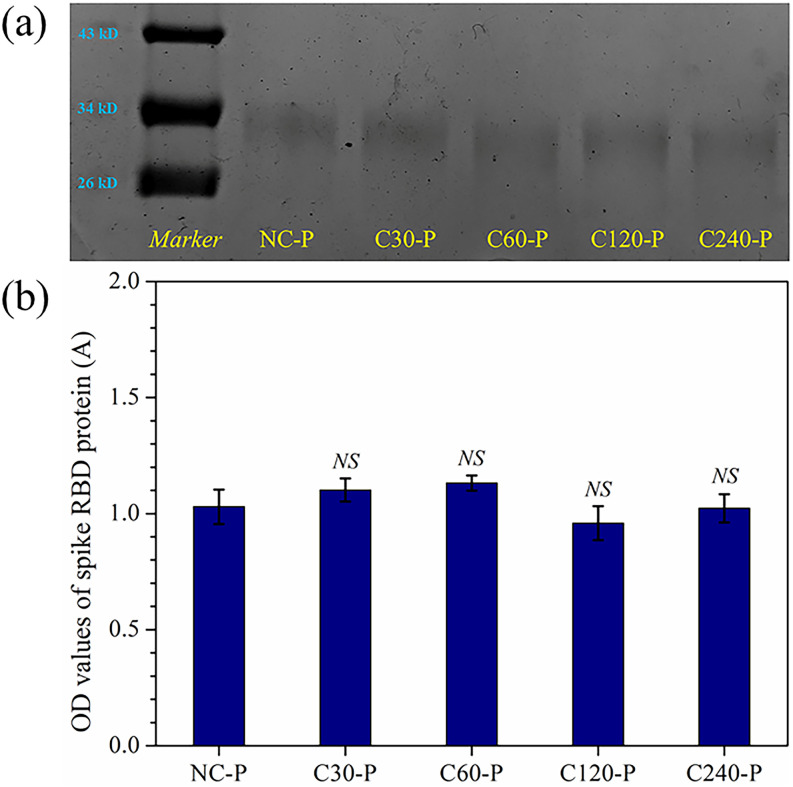
One dimensional SDS-PAGE image (a) and ELISA results (b) of spike RBD protein in NC-P, C30-P, C60-P, C120-P, and C240-P groups. “*NS*” represents no significant difference as compared with the NC-P group (p > 0.05).

As shown in Fig. 6(a), the first lane represented a protein marker with selected molecular bands, including 43, 34, and 26 kD. While the molecular information of spike RBD protein in NC-P, C30-P, C60-P, C120-P, and C240-P groups was listed in the rest lanes (left to right), respectively. It was obvious that the spike RBD protein only displayed single band around 34 kD with the same location, brightness, and thickness as compared with the NC-P group. In addition to SDS-PAGE, ELISA was employed to further understand the effects of CAP treatment on conformation of spike RBD protein. As shown in Fig. 6(b), the OD value of NC-P, C30-P, C60-P, C120-P, and C240-P was 1.03, 1.10, 1.13, 0.96, and 1.02 A, respectively. The significance analysis results showed that the OD values of C30-P, C60-P, C120-P, and C240-P all presented no significant difference (p > 0.05) as compared with the NC-P group.

### E. RNA changes

**FIG. 7.**
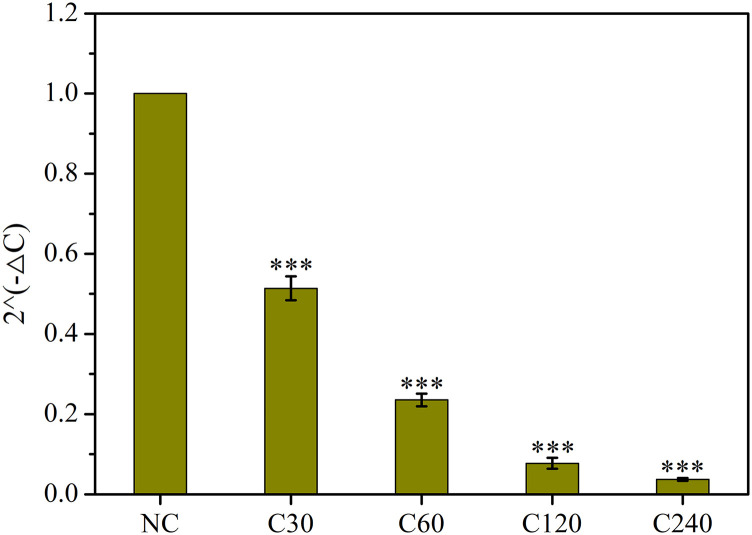
The relative concentration of RNA in NC, C30, C60, C120, and C240 groups. “***” represents extremely significant difference as compared with the NC group (p < 0.01).

As shown in Fig. 7, the RNA concentration of the NC group was set as 1, and then the values of C30, C60, C120, and C240 were 0.51, 0.24, 0.08, and 0.04, respectively. Furthermore, the relative concentration of RNA in CAP treated groups was significantly reduced (p < 0.01) as compared with the NC group.

## IV. DISCUSSION

In the past several months, though the molecular architecture, infection process, survival time, and host cell of SARS-CoV-2 virus are all unraveled and more than 100 vaccines were developed, the confirmed cases and deaths are growing every day, while the people who are administered the vaccine around the world are less than 1/3.^44–47^ While administering vaccines, adopting strategies to inhibit virus transmission is highly important so that fights for enough time for the rest people to get vaccines. Cold atmospheric plasma is regarded as a promising technology in antivirus, antifungal, and antibacterial fields with low consumption, safety, and efficient properties.^48^ Thus, the basic aim of our study is to develop an intelligent CAP environmental cleaning system, which can be widely used to eliminate SARS-CoV-2 virus in public areas such as schools, banks, hospitals, toilets, parks, buses, and metros. In this study, the antiviral ability of different CAP exposure doses on the SARS-CoV-2 like pseudovirus was studied, and the underlying mechanism is fully discussed below.

As shown above, the pseudovirus infection activity in C30, C60, C120, and C240 groups was significantly decreased as compared with the NC group. While the CAP treated pseudovirus in the C240 group displayed feeble infection ability on the 293T cell. Moreover, CAP treatment presented the inhibition effect on pseudovirus infection with the dose dependent effect. It was related to the results by Pai *et al.*^49^ and Wiegand *et al.*,^50^ who both held that cold atmospheric pressure plasma displayed dose- and time-dependent effects on different types of Mammalian cells, such as HePG2 cells.

The OES results indicated that our DBD plasma device can produce abundance of RS, such as singlet oxygen (^1^O_2_) and hydroxyl radical (OH·). This was related to previous studies, which verified that the RS produced by the low temperature air plasma system under atmospheric pressure are the same.^51–53^ In addition, the contents of RS can be adjusted by changing the discharge voltage, current, power, and time of plasma devices for a special demand.^54,55^

In order to fully understand the underlying mechanism about how CAP resulted in a significant decrease in pseudovirus infection ability on the 293T cell, the transmission electron microscope (TEM) images of pseudovirus in each group were analyzed. The TEM results showed that CAP exposure for 30 s presented no significant damage on pseudovirus and the virus cell still remained intact. However, the cell membrane of pseudovirus in C60 and C120 groups was obviously destroyed so that the uranyl acetate dye flowed into cell and appear deep black. Furthermore, CAP treatment for 240 s led to an extremely significant ruin on the cellular structure of pseudovirus. These results were related to Guo *et al.*^56^ and Filipic *et al.*,^57^ who concluded that the RS produced by plasma can interact with the peptide bond and amino acid of protein molecules, therefore causing damage to the cell membrane.

Taking account of the key important role of the spike protein in the procedure of SRAS-CoV-2 virus infection, the primary structure and conformation changes of spike RBD domain were characterized by SDS-PAGE and ELISA. The results of two experiments indicated that CAP treatment for 30, 60, 120, and 240 s have no effects on the molecular weight and conformation of spike RBD domain. The conclusion was the same as the research by Sakudo *et al.*, who reported that the viral proteins were unchanged after plasma treatment for 5 and 15 min.^58,59^ On the other hand, the results of both Wu *et al.*^60^ and Guo *et al.*^61^ presented that adjusting the discharge parameters, such as exposure time of the plasma device, can reach different even opposite results.

As compared with other research related to inhibition of SARS-CoV-2 virus transmission recently, we are curious about whether the RS can get further interacted with RNA when it enters the virus cell. Thus, the qPCR assay was conducted to detect the RNA changes of pseudovirus under different DBD plasma exposure doses. As expected, the RNA concentration of pseudovirus in C30, C60, C120, and C240 groups was significantly reduced as compared with the NC group. The results indicated that CAP treatment can destroy the RNA or main RNA polymerases, therefore reducing the total RNA concentration. This result was the same as that reported by Capelli *et al.*^62^ and Bisag *et al.*,^63^ who also said that the RS produced by plasma system can successfully degrade viral RNA, therefore suppressing the virus infection.

## V. CONCLUSION

In conclusion, our present work indicated that DBD plasma can inhibit pseudovirus infection through destroying the cell membrane and get interacted with RNA and RNA polymerases with the dose dependent effect. Our studies propose a promising strategy against stainless steel transmission of SARS-CoV-2 virus. Related research about adjusting the discharge voltage, current, power, and feed gas of the CAP system needs further research. On the other hand, the potential effects of CAP on S protein, RNA, and other compositions of SARS-CoV-2 virus should be further explored.

## Data Availability

The data that support the findings of this study are available from the corresponding authors upon reasonable request.

## References

[1] S. Kannan, P. S. S. Ali, A. Sheeza, and K. Hemalatha, “COVID-19 (novel coronavirus 2019)—Recent trends,” Eur. Rev. Med. Pharmacol. Sci. 24, 2006 (2020). 10.26355/eurrev_202002_20378 32141569

[2] S. J. Häfner, “Pandemic number five—Latest insights into the COVID-19 crisis,” Biomed. J. 43, 305–310 (2020). 10.1016/j.bj.2020.08.008 32967801 PMC7451056

[3] K. Lundstrom, “Coronavirus pandemic: Treatment and future prevention,” Future Microbiol. 15, 1507 (2020). 10.2217/fmb-2020-0174 33140657 PMC7675013

[4] Y. Zhang, S. Gargan, Y. Lu, and N. J. Stevenson, “An overview of current knowledge of deadly CoVs and their interface with innate immunity,” Viruses 13, 560 (2021). 10.3390/v13040560 33810391 PMC8066579

[5] A. A. M. Sohag, M. A. Hannan, S. Rahman, M. Hossain, M. Hasan, M. K. Khan, A. Khatun, R. Dash, and M. J. Uddin, “Revisiting potential druggable targets against SARS-CoV-2 and repurposing therapeutics under preclinical study and clinical trials: A comprehensive review,” Drug Dev. Res. 81, 919 (2020). 10.1002/ddr.21709 PMC7361641 32632960

[6] J. B. Case, A. L. Bailey, A. S. Kim, R. E. Chen, and M. S. Diamond, “Growth, detection, quantification, and inactivation of SARS-CoV-2,” Virology 548, 39–48 (2020). 10.1016/j.virol.2020.05.015 32838945 PMC7293183

[7] P. Attri, K. Koga, and M. Shiratani, “Possible impact of plasma oxidation on the structure of the C-terminal domain of SARS-CoV-2 spike protein: A computational study,” Appl. Phys. Express 14, 027002 (2021). 10.35848/1882-0786/abd717

[8] R. Noor, “Developmental status of the potential vaccines for the mitigation of the COVID-19 pandemic and a focus on the effectiveness of the Pfizer-BioNTech and Moderna mRNA vaccines,” Curr. Clin. Microbiol. Rep. (published online, 2021). 10.1007/s40588-021-00162-y PMC7927780 33686365

[9] S. Kashte, A. Gulbake, S. F. El-Amin III, and A. Gupta, “COVID-19 vaccines: Rapid development, implications, challenges and future prospects,” Hum. Cell 34, 711–733 (2021). 10.1007/s13577-021-00512-4 33677814 PMC7937046

[10] A. Fernandes, S. Chaudhari, N. Jamil, and G. Gopalakrishnan, “COVID-19 vaccine,” Endocr. Pract. 27, 170–172 (2021). 10.1016/j.eprac.2021.01.013 33515760 PMC7839427

[11] K. S. Corbett, D. K. Edwards, S. R. Leist, O. M. Abiona, S. Boyoglu-Barnum, R. A. Gillespie, S. Himansu, A. Schäfer, C. T. Ziwawo, A. T. DiPiazza, K. H. Dinnon, S. M. Elbashir, C. A. Shaw, A. Woods, E. J. Fritch, D. R. Martinez, K. W. Bock, M. Minai, B. M. Nagata, G. B. Hutchinson, K. Wu, C. Henry, K. Bahl, D. Garcia-Dominguez, L. Ma, I. Renzi, W.-P. Kong, S. D. Schmidt, L. Wang, Y. Zhang, E. Phung, L. A. Chang, R. J. Loomis, N. E. Altaras, E. Narayanan, M. Metkar, V. Presnyak, C. Liu, M. K. Louder, W. Shi, K. Leung, E. S. Yang, A. West, K. L. Gully, L. J. Stevens, N. Wang, D. Wrapp, N. A. Doria-Rose, G. Stewart-Jones, H. Bennett, G. S. Alvarado, M. C. Nason, T. J. Ruckwardt, J. S. McLellan, M. R. Denison, J. D. Chappell, I. N. Moore, K. M. Morabito, J. R. Mascola, R. S. Baric, A. Carfi, and B. S. Graham, “SARS-CoV-2 mRNA vaccine design enabled by prototype pathogen preparedness,” Nature 586, 567–571 (2020). 10.1038/s41586-020-2622-0 32756549 PMC7581537

[12] S. H. Shahcheraghi, J. Ayatollahi, A. A. Aljabali, M. D. Shastri, S. D. Shukla, D. K. Chellappan, N. K. Jha, K. Anand, N. K. Katari, M. Mehta, S. Satija, H. Dureja, V. Mishra, A. G. Almutary, A. M. Alnuqaydan, N. Charbe, P. Prasher, G. Gupta, K. Dua, M. Lotfi, H. A. Bakshi, and M. M. Tambuwala, “An overview of vaccine development for COVID-19,” Ther. Delivery 12, 235 (2021). 10.4155/tde-2020-0129 PMC7923686 33624533

[13] E. U. Haq, J. Yu, and J. Guo, “Frontiers in the COVID-19 vaccines development,” Exp. Hematol. Oncol. 9, 24 (2020). 10.1186/s40164-020-00180-4 32901214 PMC7470435

[14] M. E. Rebeaud and F. Zores, “SARS-CoV-2 and the use of chloroquine as an antiviral treatment,” Front. Med. 7, 184 (2020). 10.3389/fmed.2020.00184 PMC7193267 32391371

[15] M. Kandeel, A. H. M. Abdelrahman, K. Oh-Hashi, A. Ibrahim, K. N. Venugopala, M. A. Morsy, and M. A. A. Ibrahim, “Repurposing of FDA-approved antivirals, antibiotics, anthelmintics, antioxidants, and cell protectives against SARS-CoV-2 papain-like protease,” J. Biomol. Struct. Dyn. (published online, 2021). 10.1080/07391102.2020.1784291 PMC7332862 32597315

[16] M. J. Hossain and S. M. A. Rahman, “Repurposing therapeutic agents against SARS-CoV-2 infection: Most promising and neoteric progress,” Expert Rev. Anti-Infect. Ther. 19, 1009 (2020). 10.1080/14787210.2021.1864327 33355520

[17] M. S. Cohen, “Hydroxychloroquine for the prevention of covid-19—Searching for evidence,” N. Engl. J. Med. 383, 585–586 (2020). 10.1056/nejme2020388 32492298 PMC7289275

[18] N. Borcherding, Y. Jethava, and P. Vikas, “Repurposing anti-cancer drugs for COVID-19 treatment,” Drug Des., Dev. Ther. 14, 5045–5058 (2020). 10.2147/dddt.s282252 PMC7680713 33239864

[19] H. Tegally, E. Wilkinson, M. Giovanetti, A. Iranzadeh, V. Fonseca, J. Giandhari, D. Doolabh, S. Pillay, E. J. San, N. Msomi, K. Mlisana, A. von Gottberg, S. Walaza, M. Allam, A. Ismail, T. Mohale, A. J. Glass, S. Engelbrecht, G. Van Zyl, W. Preiser, F. Petruccione, A. Sigal, D. Hardie, G. Marais, N.-y. Hsiao, S. Korsman, M.-A. Davies, L. Tyers, I. Mudau, D. York, C. Maslo, D. Goedhals, S. Abrahams, O. Laguda-Akingba, A. Alisoltani-Dehkordi, A. Godzik, C. K. Wibmer, B. T. Sewell, J. Lourenço, L. C. J. Alcantara, S. L. Kosakovsky Pond, S. Weaver, D. Martin, R. J. Lessells, J. N. Bhiman, C. Williamson, and T. de Oliveira, “Detection of a SARS-CoV-2 variant of concern in South Africa,” Nature 592, 438–443 (2021). 10.1038/s41586-021-03402-9 33690265

[20] F. Novazzi, A. Baj, A. Genoni, P. G. Spezia, A. Colombo, G. Cassani, C. Zago, R. Pasciuta, D. D. Gasperina, C. Zago, W. Ageno, P. Severgnini, F. Dentali, D. Focosi, and F. Maggi, “SARS-CoV-2 B.1.1.7 reinfection after previous COVID-19 in two immunocompetent Italian patients,” J Med Virol. (published online, 2021). 10.1002/jmv.27066 PMC8242781 33969504

[21] L. Bian, F. Gao, J. Zhang, Q. He, Q. Mao, M. Xu, and Z. Liang, “Effects of SARS-CoV-2 variants on vaccine efficacy and response strategies,” Expert Rev. Vaccines 20, 365 (2021). 10.1080/14760584.2021.1903879 33851875 PMC8054487

[22] H. Kanamori, “Rethinking environmental contamination and fomite transmission of SARS-CoV-2 in the healthcare,” J. Infect. 82, e17–e18 (2021). 10.1016/j.jinf.2020.08.041 PMC7450250 32860816

[23] E. Hoseinzadeh, J. Safoura, M. Farzadkia, F. Mohammadi, H. Hossini, and M. Taghavi, “An updated min-review on environmental route of the SARS-CoV-2 transmission,” Ecotoxicol. Environ. Saf. 202, 111015 (2020). 10.1016/j.ecoenv.2020.111015 32800237 PMC7346818

[24] L. Xie, F. Liu, J. Liu, and H. Zeng, “A nanomechanical study on deciphering the stickiness of SARS-CoV-2 on inanimate surfaces,” ACS Appl. Mater. Interfaces 12, 58360–58368 (2020). 10.1021/acsami.0c16800 33337873

[25] E. A. Meyerowitz, A. Richterman, R. T. Gandhi, and P. E. Sax, “Transmission of SARS-CoV-2: A review of viral, host, and environmental factors,” Ann. Intern. Med. 174, 69–79 (2021). 10.7326/m20-5008 32941052 PMC7505025

[26] F. Carraturo, C. Del Giudice, M. Morelli, V. Cerullo, G. Libralato, E. Galdiero, and M. Guida, “Persistence of SARS-CoV-2 in the environment and COVID-19 transmission risk from environmental matrices and surfaces,” Environ. Pollut. 265, 115010 (2020). 10.1016/j.envpol.2020.115010 32570023 PMC7280109

[27] V. Senatore, T. Zarra, A. Buonerba, K.-H. Choo, S. W. Hasan, G. Korshin, C.-W. Li, M. Ksibi, V. Belgiorno, and V. Naddeo, “Indoor versus outdoor transmission of SARS-COV-2: Environmental factors in virus spread and underestimated sources of risk,” Euro-Mediterr. Environ. Integr. 6, 30 (2021). 10.1007/s41207-021-00243-w PMC7873670 33585671

[28] X. Li, Q. Wang, P. Ding, Y. Cha, Y. Mao, C. Ding, W. Gu, Y. Wang, B. Ying, X. Zhao, L. Pan, Y. Li, J. Chang, C. Meng, J. Zhou, Z. Tang, R. Sun, F. Deng, C. Wang, L. Li, J. Wang, C. R. MacIntyre, Z. Wu, Z. Feng, S. Tang, and D. Xu, “Risk factors and on-site simulation of environmental transmission of SARS-CoV-2 in the largest wholesale market of Beijing, China,” Sci. Total Environ. 778, 146040 (2021). 10.1016/j.scitotenv.2021.146040 33711597 PMC7921786

[29] S. Kumar, R. Singh, N. Kumari, S. Karmakar, M. Behera, A. J. Siddiqui, V. D. Rajput, T. Minkina, K. Bauddh, and N. Kumar, “Current understanding of the influence of environmental factors on SARS-CoV-2 transmission, persistence, and infectivity,” Environ. Sci. Pollut. Res. Int. 28, 6267–6288 (2021). 10.1007/s11356-020-12165-1 33387315 PMC7776306

[30] C. Tan, C. Gao, Q. Zhou, W. Van Driel, H. Ye, and G. Zhang, “The inactivation mechanism of chemical disinfection against SARS-CoV-2: From MD and DFT perspectives,” RSC Adv. 10, 40480–40488 (2020). 10.1039/d0ra06730j PMC9057723 35520849

[31] E. Criscuolo, R. A. Diotti, R. Ferrarese, C. Alippi, G. Viscardi, C. Signorelli, N. Mancini, M. Clementi, and N. Clementi, “Fast inactivation of SARS-CoV-2 by UV-C and ozone exposure on different materials,” Emerging Microbes Infect. 10, 206–210 (2021). 10.1080/22221751.2021.1872354 PMC7872580 33399524

[32] J. Biryukov, J. A. Boydston, R. A. Dunning, J. J. Yeager, S. Wood, A. Ferris, D. Miller, W. Weaver, N. E. Zeitouni, D. Freeburger, P. Dabisch, V. Wahl, M. C. Hevey, and L. A. Altamura, “SARS-CoV-2 is rapidly inactivated at high temperature,” Environ. Chem. Lett. 19, 1773 (2021). 10.1007/s10311-021-01187-x PMC7856623 33551702

[33] R. B. Martins, I. A. Castro, M. Pontelli, J. P. Souza, T. M. Lima, S. R. Melo, J. P. Z. Siqueira, M. H. Caetano, E. Arruda, and M. T. G. de Almeida, “SARS-CoV-2 inactivation by ozonated water: A preliminary alternative for environmental disinfection,” Ozone: Sci. Eng. 43, 108–111 (2020). 10.1080/01919512.2020.1842998

[34] R. Jain, P. Sarkale, D. Mali, A. M. Shete, D. Y. Patil, T. Majumdar, A. Suryawanshi, S. Patil, S. Mohandas, and P. D. Yadav, “Inactivation of SARS-CoV-2 by gamma irradiation,” Indian J. Med. Res. 153, 196 (2021). 10.4103/ijmr.IJMR_2789_20 33818476 PMC8184089

[35] A. Gidari, S. Sabbatini, S. Bastianelli, S. Pierucci, C. Busti, D. Bartolini, A. M. Stabile, C. Monari, F. Galli, M. Rende, G. Cruciani, and D. Francisci, “SARS-CoV-2 survival on surfaces and the effect of UV-C light,” Viruses 13, 408 (2021). 10.3390/v13030408 33807521 PMC7998261

[36] J. Burton, H. Love, K. Richards, C. Burton, S. Summers, J. Pitman, L. Easterbrook, K. Davies, P. Spencer, M. Killip, P. Cane, C. Bruce, and A. D. G. Roberts, “The effect of heat-treatment on SARS-CoV-2 viability and detection,” J. Virol. Methods 290, 114087 (2021). 10.1016/j.jviromet.2021.114087 33515663 PMC7840429

[37] C. Ma, A. Nikiforov, N. De Geyter, X. Dai, R. Morent, and K. K. Ostrikov, “Future antiviral polymers by plasma processing,” Prog. Polym. Sci. 118, 101410 (2021). 10.1016/j.progpolymsci.2021.101410 33967350 PMC8085113

[38] L. Lin, C.-B. Ding, T. Jin, X.-H. Han, H. Zhou, Z.-W. Wu, and Y.-Y. Pan, “A meaningful attempt: Applying dielectric barrier discharge plasma to induce apoptosis of MDA-MB-231 cells via regulating HIF-1α/VEGFA expression,” Surf. Coat. Technol. 401, 126293 (2020). 10.1016/j.surfcoat.2020.126293

[39] S. Hu, X. Ye, Q. Cai, W. Xi, J. Shen, Y. Lan, Z. Ye, C. Ye, Y. Zhang, Z. Xu, and C. Cheng, “Study on inactivation effects and regeneration inhibition mechanism of atmospheric-pressure helium plasma jet on *Acinetobacter baumannii* biofilm,” IEEE Trans. Plasma Sci. 49, 307–316 (2021). 10.1109/tps.2020.3042809

[40] C. Ding, P. Huang, L. Feng, T. Jin, Y. Zhou, Y. He, Z. Wu, and Y. Liu, “Immediate intervention effect of dielectric barrier discharge on acute inflammation in rabbit’s ear wound,” AIP Adv. 10, 025008 (2020). 10.1063/1.5139953

[41] Q. Li, Q. Liu, W. Huang, X. Li, and Y. Wang, “Current status on the development of pseudoviruses for enveloped viruses,” Rev. Med. Virol. 28, e1963 (2018). 10.1002/rmv.1963 PMC7169153 29218769

[42] Y. Huang, C. Yang, X.-f. Xu, W. Xu, and S.-w. Liu, “Structural and functional properties of SARS-CoV-2 spike protein: Potential antivirus drug development for COVID-19,” Acta Pharmacol. Sin. 41, 1141–1149 (2020). 10.1038/s41401-020-0485-4 32747721 PMC7396720

[43] B. Hu, H. Guo, P. Zhou, and Z.-L. Shi, “Characteristics of SARS-CoV-2 and COVID-19,” Nat. Rev. Microbiol. 19, 141–154 (2021). 10.1038/s41579-020-00459-7 33024307 PMC7537588

[44] H. Yao, Y. Song, Y. Chen, N. Wu, J. Xu, C. Sun, J. Zhang, T. Weng, Z. Zhang, Z. Wu, L. Cheng, D. Shi, X. Lu, J. Lei, M. Crispin, Y. Shi, L. Li, and S. Li, “Molecular architecture of the SARS-CoV-2 virus,” Cell 183, 730–738 e713 (2020). 10.1016/j.cell.2020.09.018 32979942 PMC7474903

[45] G. Yan, Y. Liming, H. Yucen, L. Fengjiang, Z. Yao, C. Lin, W. Tao, S. Qianqian, M. Zhenhua, Z. Lianqi, G. Ji, Z. Litao, Z. Ying, W. Haofeng, Z. Yan, Z. Chen, H. Tianyu, H. Tian, Z. Bing, Y. Xiuna, L. Jun, Y. Haitao, L. Zhijie, X. Wenqing, L. W. Guddat, W. Quan, L. Zhiyong, and R. Zihe, “Structure of the RNA-dependent RNA polymerase from COVID-19 virus,” Science 368, 779 (2020). 10.1126/science.abb7498 32277040 PMC7164392

[46] Q. Wang, J. Wu, H. Wang, Y. Gao, Q. Liu, A. Mu, W. Ji, L. Yan, Y. Zhu, C. Zhu, X. Fang, X. Yang, Y. Huang, H. Gao, F. Liu, J. Ge, Q. Sun, X. Yang, W. Xu, Z. Liu, H. Yang, Z. Lou, B. Jiang, L. W. Guddat, P. Gong, and Z. Rao, “Structural basis for RNA replication by the SARS-CoV-2 polymerase,” Cell 182, 417–428 e413 (2020). 10.1016/j.cell.2020.05.034 32526208 PMC7242921

[47] Z. Jin, X. Du, Y. Xu, Y. Deng, M. Liu, Y. Zhao, B. Zhang, X. Li, L. Zhang, C. Peng, Y. Duan, J. Yu, L. Wang, K. Yang, F. Liu, R. Jiang, X. Yang, T. You, X. Liu, X. Yang, F. Bai, H. Liu, X. Liu, L. W. Guddat, W. Xu, G. Xiao, C. Qin, Z. Shi, H. Jiang, Z. Rao, and H. Yang, “Structure of M^pro^ from SARS-CoV-2 and discovery of its inhibitors,” Nature 582, 289–293 (2020). 10.1038/s41586-020-2223-y 32272481

[48] D. Braný, D. Dvorská, E. Halašová, and H. Škovierová, “Cold atmospheric plasma: A powerful tool for modern medicine,” Int. J. Mol. Sci. 21, 2932 (2020). 10.3390/ijms21082932 PMC7215620 32331263

[49] K. K. Pai, K. Singarapu, J. D. Jacob, and S. V. Madihally, “Dose dependent selectivity and response of different types of mammalian cells to surface dielectric barrier discharge (SDBD) plasma,” Plasma Processes Polym. 12, 666–677 (2015). 10.1002/ppap.201400134

[50] C. Wiegand, S. Fink, O. Beier, K. Horn, A. Pfuch, A. Schimanski, B. Grünler, U.-C. Hipler, and P. Elsner, “Dose- and time-dependent cellular effects of cold atmospheric pressure plasma evaluated in 3D skin models,” Skin Pharmacol. Physiol. 29, 257–265 (2016). 10.1159/000450889 27811481

[51] Y. Zhong-Cai, S. H. I. Jia-Ming, C. Zong-Sheng, and X. U. Bo, “Electrical and spectral property of cold arc plasma at atmospheric pressure,” J. Plasma Phys. 78, 617–620 (2012). 10.1017/s0022377812000463

[52] Q. Xiong, X. Lu, J. Liu, Y. Xian, Z. Xiong, F. Zou, C. Zou, W. Gong, J. Hu, K. Chen, X. Pei, Z. Jiang, and Y. Pan, “Temporal and spatial resolved optical emission behaviors of a cold atmospheric pressure plasma jet,” J. Appl. Phys. 106, 083302 (2009). 10.1063/1.3239512

[53] S. Pavasupree, N. Chanchula, A. Bootchanont, C. Wattanawikkam, P. Jitjing, D. Boonyawan, and P. Porjai, “Enhancement propagation of protocorms in orchid (*Cymbidium tracyanum* L. Castle) by cold atmospheric pressure air plasma jet,” Plasma Chem. Plasma Process. 41, 573–589 (2021). 10.1007/s11090-020-10148-1

[54] B. B. Sahu, S. B. Jin, and J. G. Han, “Development and characterization of a multi-electrode cold atmospheric pressure DBD plasma jet aiming plasma application,” J. Anal. At. Spectrom. 32, 782–795 (2017). 10.1039/c6ja00419a

[55] M. Mateu-Sanz, J. Tornin, M. P. Ginebra, and C. Canal, “Cold atmospheric plasma: A new strategy based primarily on oxidative stress for osteosarcoma therapy,” J. Clin. Med. 10, 893 (2021). 10.3390/jcm10040893 33672274 PMC7926371

[56] L. Guo, R. Xu, L. Gou, Z. Liu, Y. Zhao, D. Liu, L. Zhang, H. Chen, and M. G. Kong, “Mechanism of virus inactivation by cold atmospheric-pressure plasma and plasma-activated water,” Appl. Environ. Microbiol. 84, 10 (2018). 10.1128/AEM.00726-18 PMC6102979 29915117

[57] A. Filipic, I. Gutierrez-Aguirre, G. Primc, M. Mozetic, and D. Dobnik, “Cold plasma, a new hope in the field of virus inactivation,” Trends Biotechnol. 38, 1278–1291 (2020). 10.1016/j.tibtech.2020.04.003 32418663 PMC7164895

[58] A. Sakudo, Y. Toyokawa, Y. Imanishi, and T. Murakami, “Crucial roles of reactive chemical species in modification of respiratory syncytial virus by nitrogen gas plasma,” Mater. Sci. Eng., C 74, 131–136 (2017). 10.1016/j.msec.2017.02.007 28254277

[59] A. Sakudo, Y. Toyokawa, and Y. Imanishi, “Nitrogen gas plasma generated by a static induction thyristor as a pulsed power supply inactivates adenovirus,” PLoS One 11, e0157922 (2016). 10.1371/journal.pone.0157922 27322066 PMC4913946

[60] Y. Wu, J. Liu, L. Gao, Y. Ma, G. Xu, X. Li, Y. Hao, X. Shi, and G.-J. Zhang, “Helium low temperature plasma induced HepG2 cells autophagy through ROS-mediated PI3K/AKT/mTOR/P70s6k signaling pathway,” AIP Adv. 9, 095034 (2019). 10.1063/1.5116292

[61] L. Guo, Z. Yao, L. Yang, H. Zhang, Y. Qi, L. Gou, W. Xi, D. Liu, L. Zhang, Y. Cheng, X. Wang, M. Rong, H. Chen, and M. G. Kong, “Plasma-activated water: An alternative disinfectant for S protein inactivation to prevent SARS-CoV-2 infection,” Chem. Eng. J. 421, 127742 (2020). 10.1016/j.cej.2020.127742 33235538 PMC7677677

[62] F. Capelli, S. Tappi, T. Gritti, A. C. de Aguiar Saldanha Pinheiro, R. Laurita, U. Tylewicz, F. Spataro, G. Braschi, R. Lanciotti, F. Gómez Galindo, V. Siracusa, S. Romani, M. Gherardi, V. Colombo, V. Sambri, and P. Rocculi, “Decontamination of food packages from SARS-CoV-2 RNA with a cold plasma-assisted system,” Appl. Sci. 11, 4177 (2021). 10.3390/app11094177

[63] A. Bisag, P. Isabelli, R. Laurita, C. Bucci, F. Capelli, G. Dirani, M. Gherardi, G. Laghi, A. Paglianti, V. Sambri, and V. Colombo, “Cold atmospheric plasma inactivation of aerosolized microdroplets containing bacteria and purified SARS-CoV-2 RNA to contrast airborne indoor transmission,” Plasma Processes Polym. 17, 2000154 (2020). 10.1002/ppap.202000154

